# Let-7c inhibits migration and epithelial–mesenchymal transition in head and neck squamous cell carcinoma by targeting *IGF1R* and *HMGA2*

**DOI:** 10.18632/oncotarget.23826

**Published:** 2018-01-02

**Authors:** Bo Hou, Hajime Ishinaga, Kaoru Midorikawa, Satoshi Nakamura, Yusuke Hiraku, Shinji Oikawa, Ning Ma, Kazuhiko Takeuchi, Mariko Murata

**Affiliations:** ^1^ Department of Otolaryngology-Head and Neck Surgery, Graduate School of Medicine, Mie University, Tsu, Mie 514-8507, Japan; ^2^ Department of Environmental and Molecular Medicine, Graduate School of Medicine, Mie University, Tsu, Mie 514-8507, Japan; ^3^ Graduate School of Health Science, Suzuka University of Medical Science, Suzuka, Mie 513-8670, Japan

**Keywords:** Let-7c, IGF1R, HMGA2, epithelial–mesenchymal transition, head and neck squamous cell carcinoma

## Abstract

To elucidate the molecular mechanisms underlying the progression of head and neck squamous cell carcinoma (HNSCC), we investigated the function of let-7c as a tumor suppressor. Let-7c expression was significantly down-regulated in HNSCC tumor tissues and cell lines. *In vitro* and *in vivo* studies revealed that let-7c negatively regulated HNSCC proliferation, migration and epithelial–mesenchymal transition (EMT). To explore the underlying mechanisms that affect these molecular events achieved by let-7c, we predicted its target genes. We performed luciferase assay and confirmed that insulin-like growth factor 1 receptor (*IGF1R*) and high mobility group AT-hook 2 (*HMGA2*) were the direct targets of let-7c. Knocking down of *IGF1R* and *HMGA2* inhibited HNSCC progression, including proliferation, migration and EMT in HNSCC cells. Re-expression of these genes overcame let-7c–mediated inhibition. Taken together, our finding suggests that let-7c inhibits HNSCC progression by targeting *IGF1R* and *HMGA2* and might be a novel target for HNSCC treatment.

## INTRODUCTION

Head and neck cancer is one of the most common types of human cancer, with nearly 690,000 new cases and 380,000 deaths occurring each year [1]. Approximately 90% of these tumors are head and neck squamous cell carcinoma (HNSCC) [2]. The therapy for HNSCC usually includes surgery with or without concurrent chemotherapy and radiation therapy, or radiation alone. Despite the use of advanced surgical techniques and effective chemotherapeutic agents, the overall survival of patients with HNSCC has remained unchanged, with a 5-year survival rate of about 50% [3]. Metastasis accounts for the majority of deaths of patients with HNSCC [4]. To date, the molecular mechanisms that promote HNSCC proliferation and migration are largely unknown. There is an urgent need to identify target molecules for novel therapeutic strategies.

MicroRNAs (miRNAs) are small non-coding RNAs that play an important regulatory role in cancer development and progression [5]. MiRNAs usually contribute to the regulation of their target genes’ mRNA by base-pairing to the 3′-untranslated region (3′-UTR), which results in either mRNA degradation or translational inhibition. It has been reported that a given miRNA can bind to mRNAs derived from hundreds of different genes [6], and it is therefore estimated that more than 30% of all protein-coding genes within the human genome are targeted by miRNAs [7]. MiRNAs can act either as oncogenes or tumor suppressors depending on the function of their target molecules and on the regulation of downstream signaling pathways [8].

The let-7 family of miRNAs is widely viewed as playing a role in tumor suppression. Humans have 10 mature let-7 family sequences (let-7a, 7b, 7c, 7d, 7e, 7f, 7g, 7i, miR-98, and miR-202). To predict which miRNA in the let-7 family is most important in terms of HNSCC progression, we first checked their expressions in HNSCC using starBase v2.0. The database generated by 37 independent studies [9] showed that let-7c was the most down-regulated miRNA (fold change = 0.236, *P* = 0.0000) in HNSCC compared to normal tissues among the 10 mature let-7 family members (let-7a, fold change = 0.760, *P* = 0.0002; let-7b, fold change = 0.847, *P* = 0.0311; let-7d, fold change = 1.388, *P* = 0.0000; let-7e, fold change = 1.087, *P* = 0.3605; let-7f, fold change = 0.774, *P* = 0.0118; let-7g, fold change = 0.858, *P* = 0.0226; let-7i, fold change = 1.230, *P* = 0.0005; miR-98, fold change = 1.0420, *P* = 0.5318; miR-202, fold change = 0.957, *P* = 0.0041). In our previous study, let-7c miRNA was demonstrated to be the only let-7 family member which showed significant difference between four paired HNSCC tissues and adjacent non-tumor tissues as determined by miRNA microarray and it was confirmed to be down-regulated in sixteen HNSCC tissues by quantitative real-time PCR (RT-qPCR) [10]. However, the exact role of let-7c underlying HNSCC progression remains unclear.

Online bioinformatics tools indicate that let-7c has a conserved binding site in the 3′-UTRs of many genes, including insulin-like growth factor 1 receptor (*IGF1R*), high mobility group AT-hook 2 (*HMGA2*) and others. *IGF1R* and *HMGA2* are important factors that are involved in cancer progression. In clinical settings, up-regulation of either IGF1R or HMGA2 protein has been reported to be strongly linked to poor prognosis in malignancies such as gastric cancer and breast cancer [11, 12]. More strikingly, *IGF1R* and *HMGA2* are well documented as attractive targets for anti-cancer treatment [13, 14]. Recent studies have reported that overexpression of *IGF1R* [15] and *HMGA2* [16] enhances tumor growth and migration in prostate cancer and colorectal cancer, respectively. In addition, *IGF1R* and *HMGA2* play an essential role in inducing epithelial-mesenchymal transition (EMT) in cancers [17, 18]. No reports have clarified the relation between let-7c and its potential targets, *IGF1R* and *HMGA2*, and the underlying mechanism of action of let-7c. Therefore, for the first time, we proposed and tested the hypothesis that let-7c inhibits tumor cell growth, migration, and EMT by directly targeting *IGF1R* and *HMGA2*. Our findings identified let-7c as a tumor suppressor miRNA and provided new insights into the molecular function of let-7c and its target genes *IGF1R* and *HMGA2*, suggesting new therapeutic targets in HNSCC.

## RESULTS

### Let-7c directly targets IGF1R and HMGA2 by binding to their 3′-UTRs

**Figure 1:**
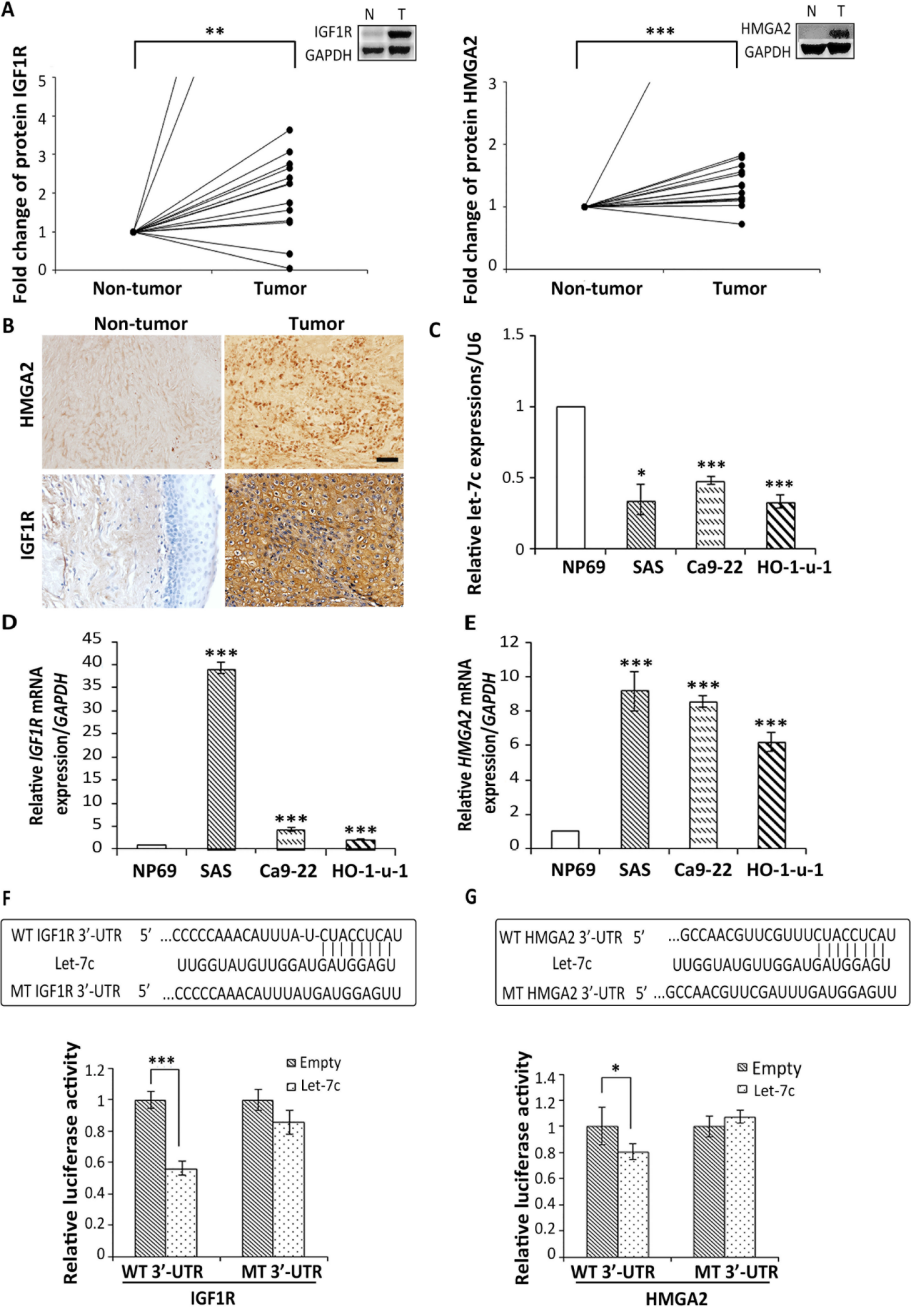
Let-7c directly binds to the 3′-UTRs of *IGF1R* and *HMGA2* Relative protein levels of (**A**) IGF1R and HMGA2. IGF1R and HMGA2 in HNSCC tumor tissues and adjacent non-tumor tissues (*n* = 15) were examined by western blot analysis with GAPDH serving as loading control. Thereafter, the target protein levels were calculated as fold change compared to paired non-tumor levels. Non-tumor levels were set as 1. Statistical analysis was performed using the Student’s paired *t*-test. (**B**) Representative IHC staining of IGF1R and HMGA2 in HNSCC tumor tissues and adjacent non-tumor tissues. Original magnification ×100 and ×200; bar represents 50 μm. Expression of (**C**) Let-7c, (**D**) *IGF1R*, and (**E**) *HMGA2* in HNSCC cell lines SAS, Ca9-22, and H0-1-u-1 compared to non-malignant nasopharyngeal epithelial cell line NP69. Let-7c-binding sites in the *IGF1R* and *HMGA2* 3′-UTRs were predicted by TargetScan. (**F**) 2619–2626nt of the *IGF1R* 3′-UTR sequence and (**G**) 21–28nt of the *HMGA2* 3′-UTR sequence, as well as the complementary let-7c binding sequences and the target mutated sequences, are shown in the boxed rectangles. For the luciferase reporter assays, SAS cells were co-transfected with 50 ng of PCMV-MIR vector or PCMV-MIR-let-7c vector and 100 ng dual-luciferase vector containing either wild-type or mutant 3′-UTR of (F) *IGF1R* and (G) *HMGA2*. The relative firefly luciferase activity normalized with renilla luciferase was measured 24 h after transfection. Statistical analysis was performed using the Student’s *t*-test. ^*^P < 0.05; ^**^P < 0.01; ^***^P < 0.001.

In our previous study, we found that let-7c expression was significantly decreased in HNSCC tissues compared to adjacent non-tumor tissues [10]. Predicting the target genes of let-7c was based on TargetScan Human online database (Release 7.0: August 2015, http://www.targetscan.org/). Let-7c has potential binding sites at the 3′-UTRs of *IGF1R* and *HMGA2* mRNAs. Measurement of IGF1R and HMGA2 protein levels in 15 HNSCC tumor tissues and adjacent non-tumor tissues showed significantly higher levels of both proteins in the HNSCC tumor tissues than in the non-tumor tissues (Figure 1A). Furthermore, we confirmed up-regulation of both proteins in HNSCC by immunohistochemistry (IHC). Positive membrane staining of IGF1R and positive nuclear staining of HMGA2 were observed in tumor tissues, with no or weak immunoreactivity of these proteins in adjacent non-tumor tissues (Figure 1B). These results indicate that *IGF1R* and *HMGA2* might be involved in HNSCC carcinogenesis through let-7c downregulation.

In addition, we confirmed that the expression levels of let-7c in HNSCC cell lines SAS, Ca9-22, and H0-1-u-1 were significantly lower than in non-malignant nasopharyngeal epithelial cell line NP69 (Figure 1C). Furthermore, *IGF1R* and *HMGA2* were significantly up-regulated in HNSCC cell lines SAS, Ca9-22, and H0-1-u-1 compared to non-malignant cell line NP69 (Figure 1D, 1E). In order to confirm that the 3′-UTRs of the *IGF1R* and *HMGA2* mRNAs were indeed targeted by let-7c, we fused the sequences of the wild-type 3′-UTRs of *IGF1R* and *HMGA2* mRNAs, as well as mutated sequences of these genes (Figure 1F and 1G) that disrupt complementary binding of let-7c, downstream of a luciferase reporter gene. All four luciferase constructs were transfected into cells that were also co-transfected with either negative-miRNA control or let-7c. As measured by luciferase assays, the activity of the wild-type *IGF1R* luciferase construct was significantly reduced by let-7c compared to control, whereas the activity of the mutant *IGF1R* construct was unaffected (Figure 1F). These results indicate that the 3′-UTR of *IGF1R* mRNA can be complemented and targeted by let-7c. Similar results were obtained in the case of *HMGA2* (Figure 1G). These findings showed that *IGF1R* and *HMGA2* mRNA were direct targets of let-7c.

### Let-7c inhibits IGF1R and HMGA2 expression in HNSCC cell lines

**Figure 2:**
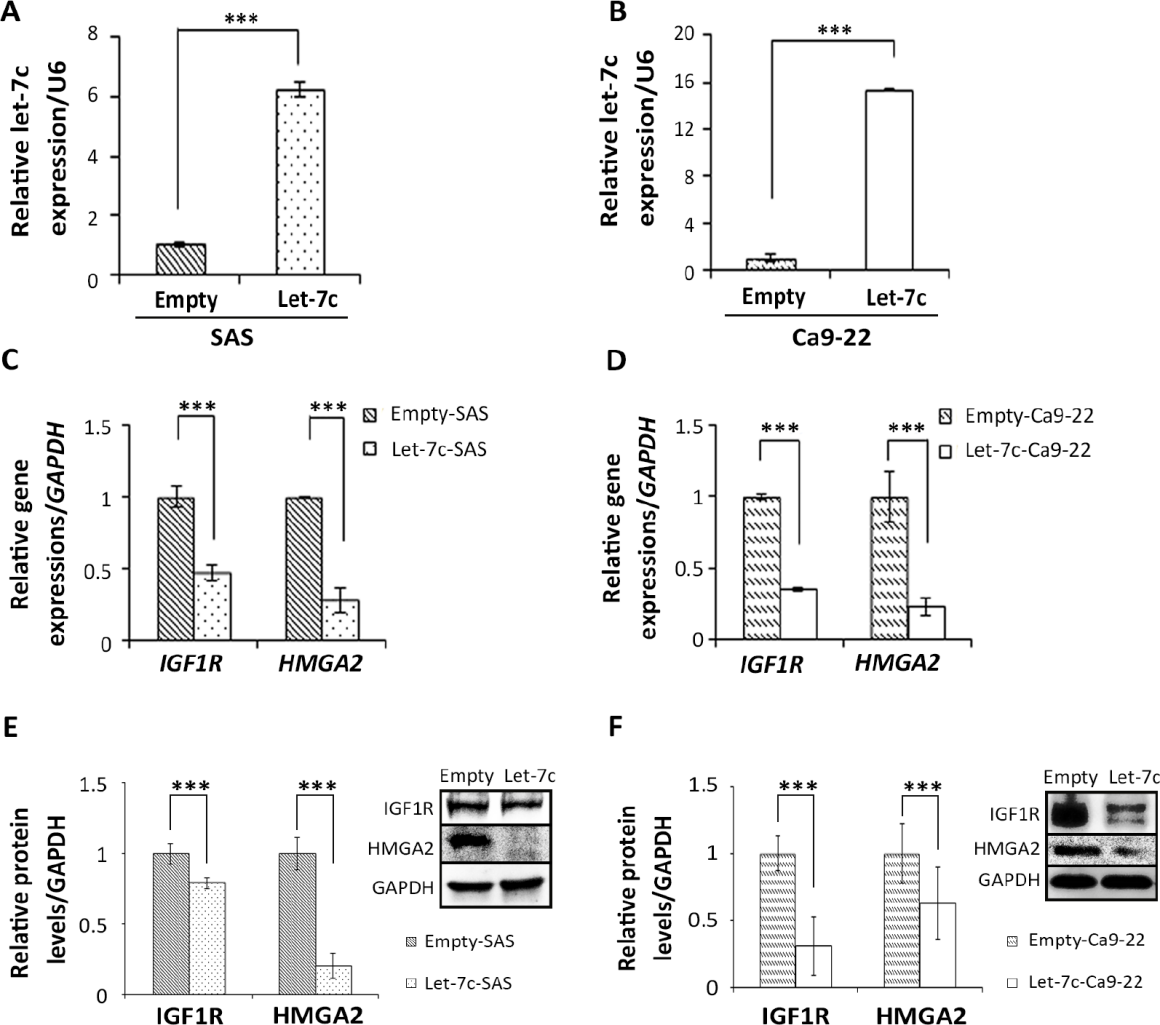
Let-7c inhibits *IGF1R* and *HMGA2* expression in HNSCC cell lines HNSCC cells were stably transfected with either pCMV-MIR vector or pCMV-MIR-let-7c vector. Expression levels of (**A, B**) let-7c and (**C, D**) *IGF1R* and *HMGA2* mRNA were examined in HNSCC cell lines SAS and Ca9-22, respectively, with RT-qPCR. (**E, F**) IGF1R and HMGA2 protein levels were examined by western blot analysis in stably transfected let-7-expressing SAS and Ca9-22 cell lines, respectively, with GAPDH serving as loading control. *P*-values were calculated using the Student’s *t*-test. ^***^P < 0.001.

Having identified *IGF1R* and *HMGA2* as targets of let-7c, we next investigated the role of let-7c in two HNSCC cell lines SAS and Ca9-22. Using the pCMV-MIR-let-7c vector, we established corresponding cell lines, SAS-let-7c and Ca9-22-let-7c, which stably expressed let-7c (Figure 2A, 2B), and examined the effect of let-7c on the expression of *IGF1R* and *HMGA2*. Overexpression of let-7c through stable transduction significantly reduced mRNA levels of *IGF1R* and *HMGA2* compared with the empty control in SAS and Ca9-22 cell lines (Figure 2C, 2D). Next, using western blotting with antibodies against IGF1R and HMGA2, we found that the levels of both proteins were also reduced in let-7c-expressing cells compared to the empty control (Figure 2E, 2F). We also determined the levels of IGF1R and HMGA2 proteins in let-7c-expressing SAS cells using immunocytochemistry (ICC), showing that let-7c significantly decreased the levels of both proteins (Supplementary Figure 1A, 1B). Of note, even transient transfection with a let-7c mimic was still sufficient to significantly elevate let-7c levels (Supplementary Figure 2A, 2B) and repress the expression of let-7c target genes, *IGF1R* and *HMGA2* (Supplementary Figure 2C, 2D), in SAS cells and Ca9-22 cells. These data suggest that *IGF1R* and *HMGA2* are repressed by let-7c in HNSCC cell lines.

### Let-7c inhibits colony formation, proliferation and migration in HNSCC cell lines

**Figure 3:**
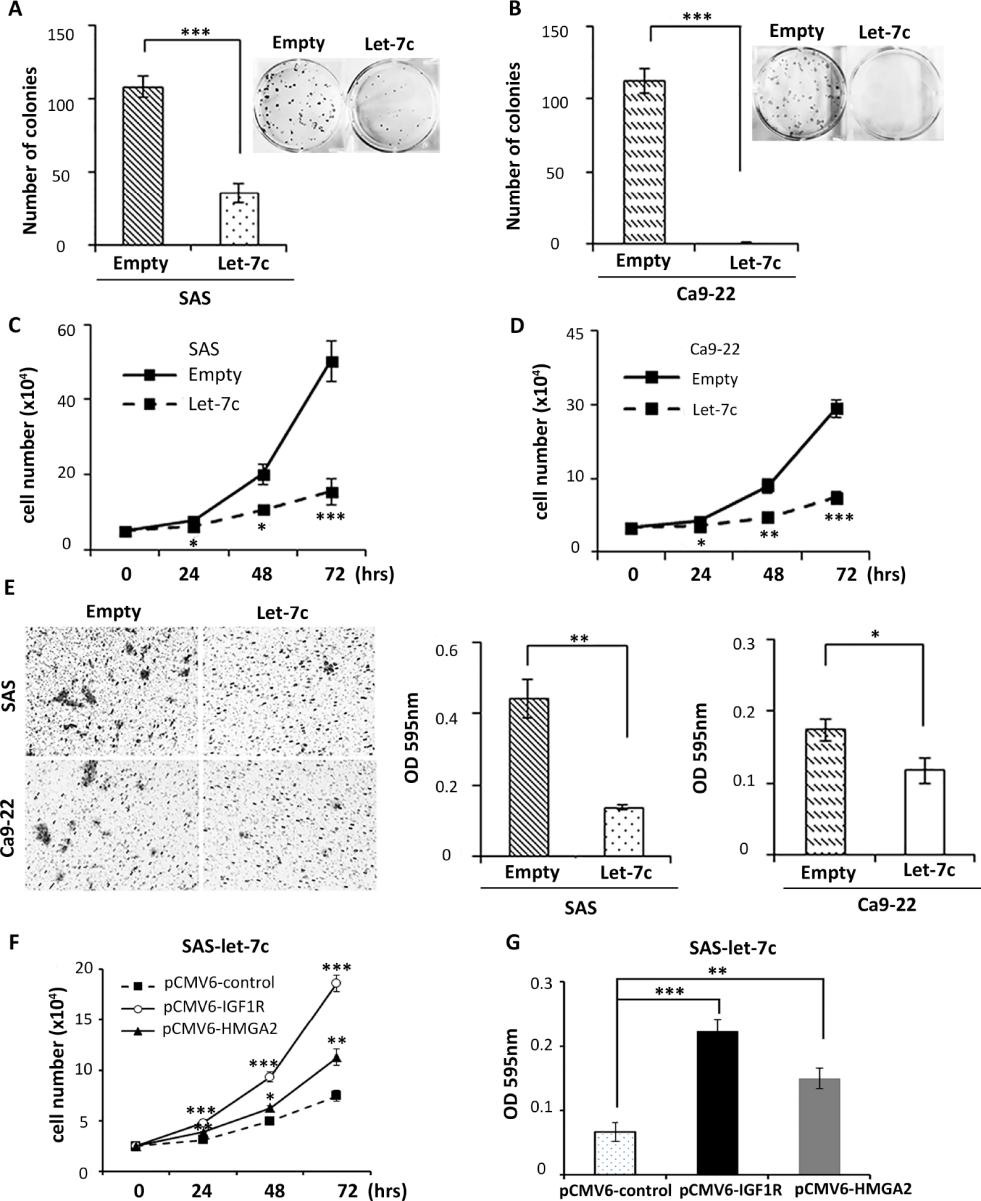
Let-7c inhibits colony formation, proliferation, and migration in HNSCC cell lines HNSCC cell lines SAS and Ca9-22 were transfected with either pCMV-MIR vector or pCMV-MIR-let-7c vector. Cells were subjected to assays of (**A, B**) colony formation, (**C, D**) proliferation, and (**E**) migration to examine growth of *in vitro* cancer cell cultures. *P*-values were calculated using the Student’s *t*-test. Following re-expression of *IGF1R* or *HMGA2* in SAS-let-7c cells using pCMV6-IGF1R or pCMV6-HMGA2, cells were subjected to assays of (**F**) proliferation and (**G**) migration. *P*-values of F and G were derived from two-way ANOVA and one-way ANOVA followed by Bonferroni correction, respectively. ^*^P < 0.05; ^**^P < 0.01; ^***^P < 0.001.

Since *IGF1R* [19] and *HMGA2* [18] were reported to promote tumorigenesis of HNSCC, we speculated that downregulation of their expression by let-7c should inhibit colony formation, proliferation, and migration of HNSCC cell lines. To validate this hypothesis, we first conducted colony formation assays in SAS and Ca9-22 cell lines. As illustrated in Figure 3A and Figure 3B, the colony number of let-7c-expressing cells was significantly smaller than that of control cells. Next, we compared the rate of cell proliferation in these two HNSCC cell lines during a 3-day incubation, and found that let-7c significantly reduced cell growth compared to the control (Figure 3C, 3D). In addition, HNSCC cells transfected with let-7c exhibited a significant defect in cell migration compared to the control (Figure 3E). To investigate whether let-7c exerted its effects by regulating *IGF1R* and *HMGA2* in HNSCC cells, we re-expressed the coding region sequence of *IGF1R* or *HMGA2* in let-7c stably transfected SAS cells (SAS-let-7c) using the pCMV6-IGF1R or pCMV6-HMGA2 plasmid, respectively. Overexpression of *IGF1R* and *HMGA2* promoted SAS-let-7c stable cell growth and migration (Figure 3F, 3G). Taken together, these results suggest that let-7c inhibits colony formation, cell proliferation, and migration in HNSCC cell lines and that re-expression of *IGF1R* or *HMGA2* overcomes let-7c–inhibited cell proliferation and migration.

### Let-7c inhibits EMT in HNSCC cell lines

**Figure 4:**
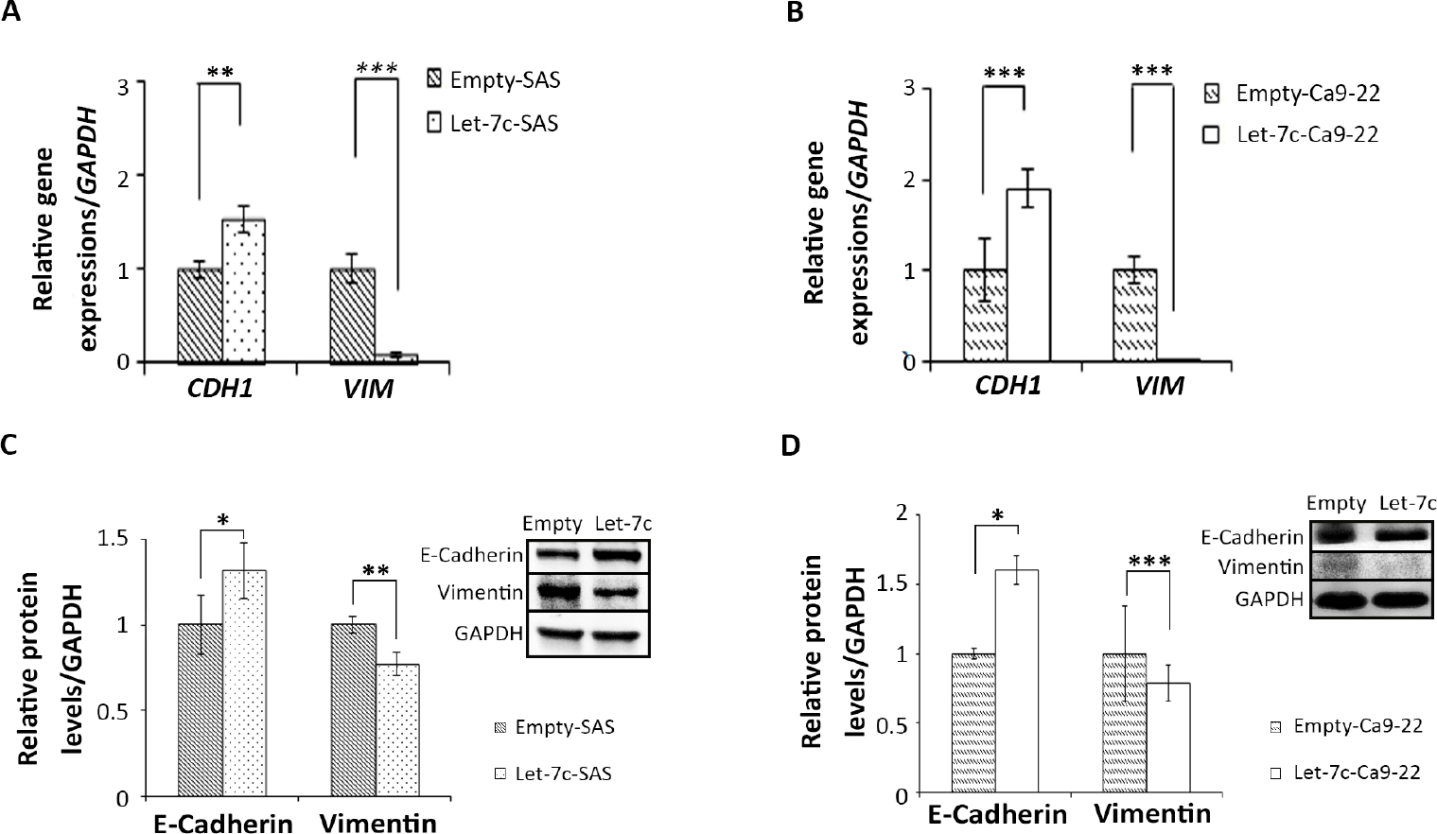
Let-7c inhibits EMT in HNSCC cell lines (**A, B**) *CDH1* and *VIM* mRNA levels in let-7c-expressing cell lines were determined by RT-qPCR, and (**C, D**) E-cadherin and vimentin protein levels were examined by western blot analysis in SAS and Ca9-22, respectively, two HNSCC cell lines stably expressing let-7c. GAPDH served as loading control. *P*-values were calculated using the Student’s *t*-test. ^*^P < 0.05; ^**^P < 0.01; ^***^P < 0.001.

Tumor cell migration is often associated with the EMT process [20, 21]. In addition, let-7c was shown to be related to EMT in non-small cell lung cancer patients [22]. We thus investigated the effects of let-7c on the EMT process in HNSCC. We examined two EMT-related molecules (E-cadherin, an epithelial marker; vimentin, a mesenchymal marker) using let-7c-expressing HNSCC cell lines. RT-qPCR revealed that the expression levels of *CDH1* were significantly higher and those of *VIM* were significantly lower in let-7c-expressing HNSCC cells than the control groups (SAS cells in Figure 4A, Ca9-22 cells in Figure 4B). Western blotting showed the same pattern in the protein levels of E-cadherin and vimentin in both cell lines (Figure 4C, 4D). ICC showed that let-7c overexpression significantly increased E-cadherin protein levels and decreased vimentin protein levels in SAS cells (Supplementary Figure 3A, 3B). In addition, SAS-empty cells were spindle-shaped mesenchymal cells, whereas SAS-let-7c cells were rounded cells with epithelial phenotypes (Supplementary Figure 3C). These data suggest that let-7c inhibits the EMT process in HNSCC cell lines.

### Let-7c inhibits tumor growth and infiltration *in vivo*

**Figure 5:**
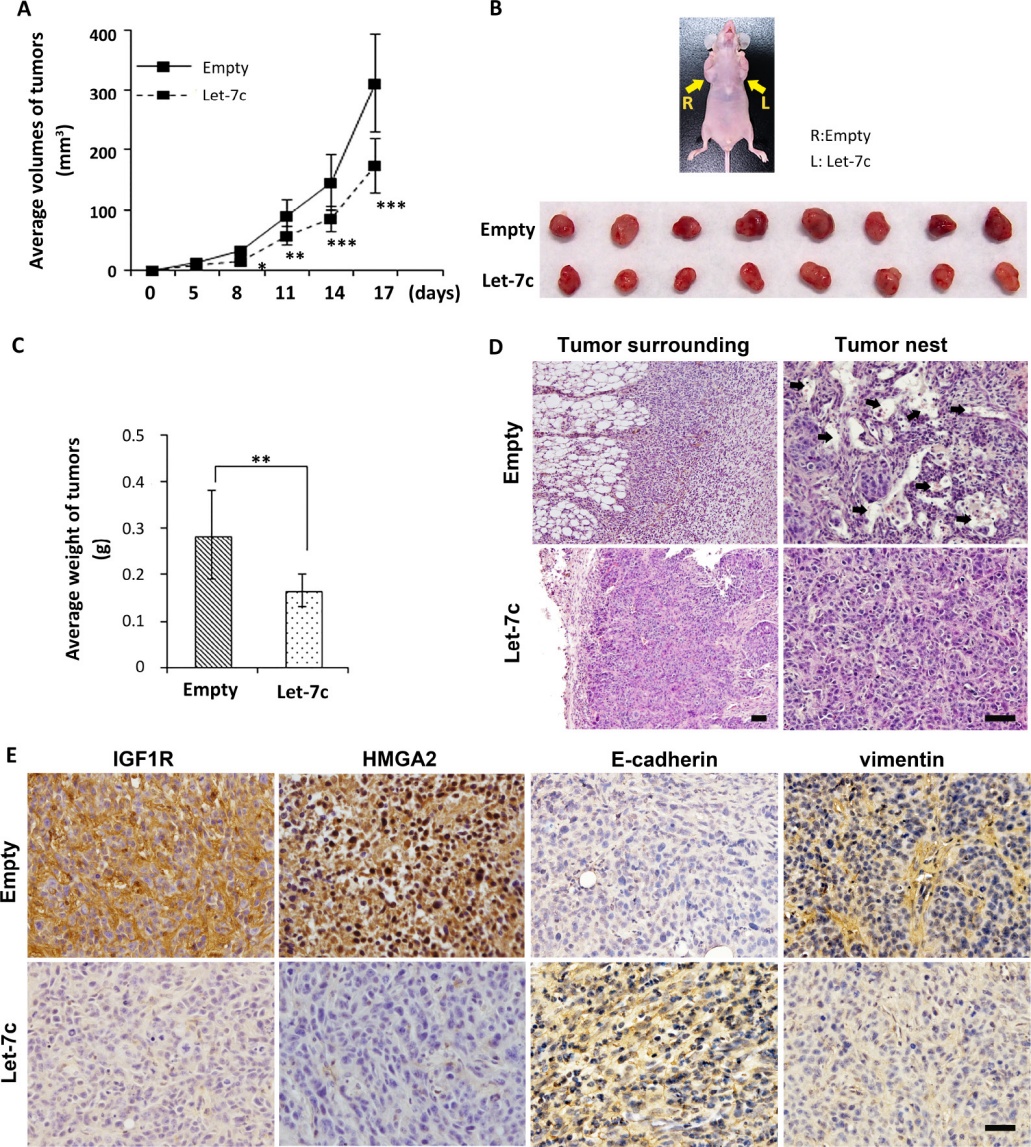
Let-7c inhibits HNSCC tumor growth and infiltration (**A**) Growth curves were drawn by measuring tumor volumes at the indicated times. (**B**) Image of subcutaneous xenografts in the mouse flanks (the arrows indicate tumor locations in a mouse injected with (L) SAS cells stably expressing let-7c or (R) control SAS cells) and excised tumors. (**C**) Weight of xenograft tumors. (**D**) H&E staining demonstrating growth properties. Arrows point to blood vessels in the cancer nests. (**E**) Representative IHC staining of IGF1R, HMGA2, E-cadherin, and vimentin in the xenograft tumors. Nuclei were counterstained with hematoxylin. Original magnification ×100 and ×200; bars represent 50 μm. *P*-values of differences between let-7c and control tumors were calculated using the Student’s paired *t*-test. ^*^P < 0.05, ^**^P < 0.01, ^***^P < 0.001.

We next aimed to investigate whether let-7c had the same properties *in vivo*. We performed a xenograft study in which let-7c–transfected SAS cells were transplanted into the flanks of BALB/c athymic nu/nu mice (*n* = 8). As illustrated in Figure 5A, tumor growth in mice injected with let-7c-expressing SAS cells was markedly slowed compared to that in mice that received control miRNA-expressing cells. The mice were sacrificed for the evaluation of tumor formation 17 days after inoculation (Figure 5B). The tumor weight of SAS xenografts was significantly lower in let-7c-expressing tumors than in control tumors (Figure 5C). The tumor xenograft experiment indicates that let-7c plays a role in suppressing HNSCC tumorigenicity.

Tumor sections were stained with H&E to visualize the morphology of the xenograft tumors. As shown in Figure 5D, tumor cells in the control group infiltrated the adipose layer of the skin around the tumor, indicating greater aggressiveness than let-7c-expressing tumors. Moreover, the number of blood vessels (Figure 5D, arrows) inside the tumor nest was much higher in the control group than in the let-7c group, indicating that let-7c inhibited infiltration of tumor cells at the invasive front *in vivo*.

To confirm that let-7c could modulate EMT phenotype *in vivo*, we investigated the protein levels of IGF1R, HMGA2, E-cadherin, and vimentin by IHC. The staining intensities of IGF1R and HMGA2 were much lower in let-7c-expressing SAS cells compared to the control. Higher levels E-cadherin and lower levels of vimentin were observed in let-7c-expressing SAS cells (Figure 5E). These results show that let-7c inhibits IGF1R and HMGA2 expression levels and reverses EMT *in vivo*.

### Silencing of IGF1R and HMGA2 potentially contributes to suppression of colony formation, cell proliferation, migration, and EMT in HNSCC cells

**Figure 6:**
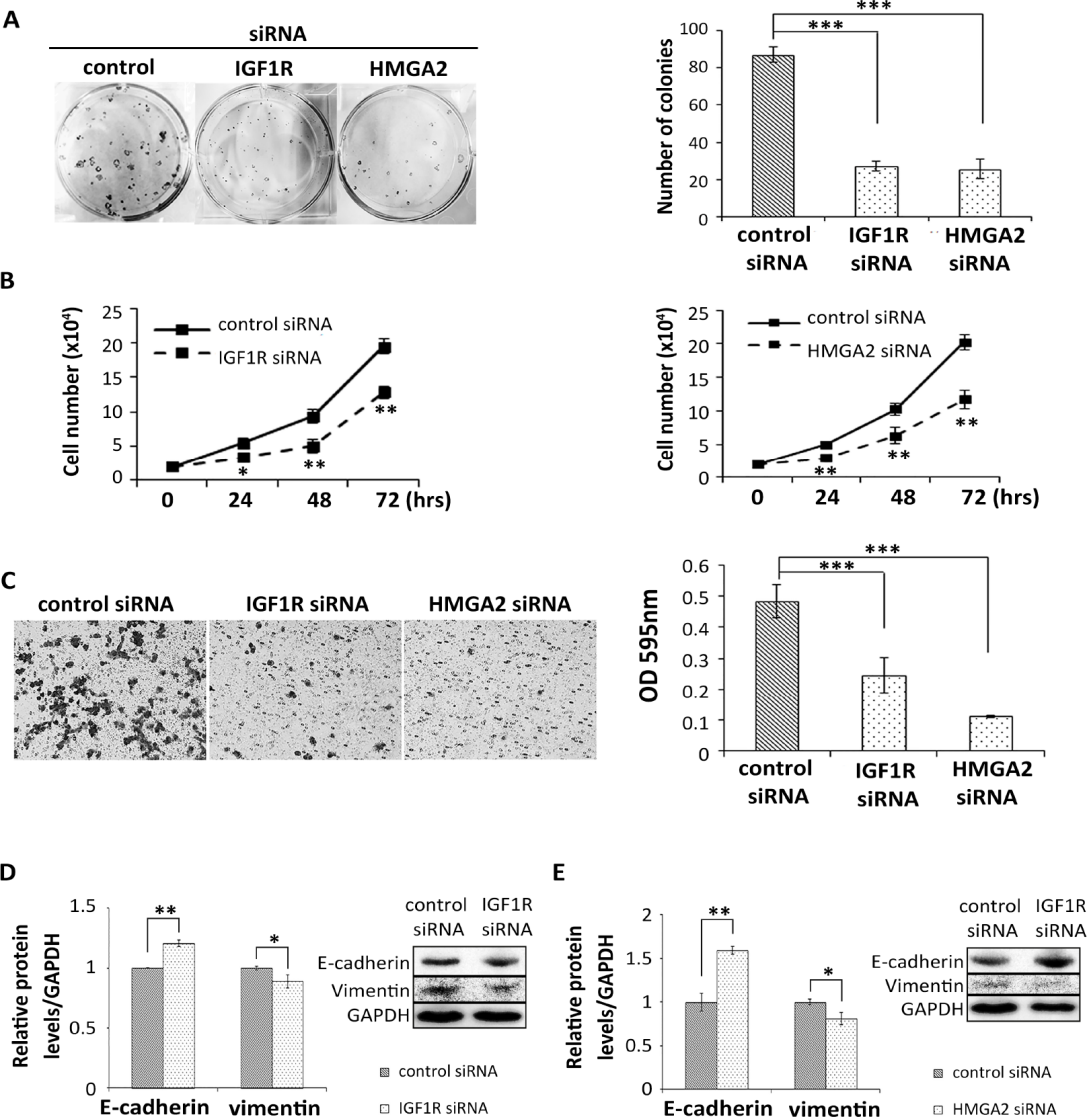
Reduction of *IGF1R* and *HMGA2* inhibits colony formation, cell proliferation, migration, and EMT in HNSCC cells *IGF1R* and *HMGA2* were knocked down using siRNAs in the HNSCC cell line SAS. Cells were subjected to assays of (**A**) colony formation, (**B**) proliferation, and (**C**) migration to examine growth of *in vitro* cancer cell cultures. (**D, E**) Levels of EMT-related proteins E-cadherin and vimentin were determined by western blot in SAS cells transfected with (D) IGF1R siRNA or (E) HMGA2. *P*-values of A and C were achieved using one-way ANOVA, followed by Bonferroni correction. (B, D, E) *P*-values were calculated using the Student’s *t*-test. ^*^P < 0.05; ^**^P < 0.01; ^***^P < 0.001.

We confirmed that mRNA and protein levels of IGF1R and HMGA2 were significantly down-regulated in SAS cells after transfection with the corresponding siRNAs (Supplementary Figure 4). We examined cell colony formation in siRNA-treated SAS cells. The number of colonies was remarkably smaller in both types of siRNA-transfected cells than in control siRNA-transfected cells (Figure 6A). In the proliferation assay, both types of siRNA-transfected cells grew significantly more slowly than control siRNA-transfected cells (Figure 6B). Migratory cells were observed less frequently in *IGF1R* and *HMGA2* siRNA-transfected cells than control siRNA-transfected cells (Figure 6C). These results suggest that reduction of *IGF1R* and *HMGA2* contributes to suppression of colony formation, cell proliferation, and migration in HNSCC cells.

To investigate the mechanism underlying cell migration, we evaluated the EMT process using *IGF1R* and *HMGA2* siRNA-transfected cells. E-cadherin protein levels were significantly up-regulated in both types of siRNA-transfected SAS cells compared to the control. In contrast, vimentin protein levels were down-regulated (Figure 6D, 6E). Taken together, these findings indicate that reduction of *IGF1R* and *HMGA2* inhibits cell migration by inducing the mesenchymal to epithelial transition process.

## DISCUSSION

Aberrant expression of miRNAs has been found in various malignant tumors. Tumor growth and metastasis are critical for tumor progression, and miRNAs are closely involved in this process through their actions on target genes. Many researchers have reported that let-7c acts as a tumor suppressor and is down-regulated during tumorigenesis of various types of cancers, including lung, colorectal and prostate cancers [23–26]. In our previous study, we found that let-7c was significantly down-regulated in HNSCC tumor tissues compared to adjacent non-tumor tissues and contributes to tumor progression [10], although HNSCCs include various tumors originating from oral cavity, hypopharynx, oropharynx, nasopharynx and larynx with difference in epidemiology, etiology and therapeutic approach. We used TargetScan Human, an online database for prediction of miRNA targets, to show that *IGF1R* and *HMGA2* were the target genes of let-7c. Western blot analysis revealed upregulation of *IGF1R* and *HMGA2* corresponding to the downregulation of let-7c in HNSCC tissues. High-risk human papillomaviruses (HPVs) have been implicated in the pathogenesis of a subset of HNSCCs, mainly arising from the oropharynx [27, 28]. To date, mounting evidence indicates that HPVs may regulate cellular miRNA expression through their E6 and E7 oncoproteins [29]. Recent studies showed that HNSCCs with IGF1R or HMGA2 expression are associated with HPV-negative status [30–32]. We checked HPV status in HNSCC patients by staining the cyclin-dependent kinase inhibitor p16, which is considered to be a biomarker for HPV-related diseases [33] (Supplementary Table 1). However, no significant differences in IGF1R and HMGA2 expression levels were observed between HPV-positive and -negative groups, with the limitation probably due to small sample size. MiRNAs can reduce gene expression by binding to the 3′-UTR of the target gene, causing mRNA degradation or suppression of translation. We demonstrated the potential binding of let-7c to the 3′-UTRs of *IGF1R* and *HMGA2* mRNAs using luciferase reporter assays. Furthermore, exogenous expression of let-7c in HNSCC cells down-regulated *IGF1R* and *HMGA2* levels both *in vitro* and *in vivo*. These results indicate that let-7c negatively regulates *IGF1R* and *HMGA2* expression by binding to the 3′-UTRs of these genes in HNSCC.

The present study demonstrated that overexpression of let-7c resulted in suppression of cell proliferation, migration, and EMT in HNSCC cells *in vitro*. EMT is critical for tumor metastasis, and high levels of let-7c have been reported to control EMT in various tumor cells [22, 34], although there are no reports specific to HNSCC. For the first time, we confirmed that overexpression of let-7c increased E-cadherin and decreased vimentin in a nude mouse xenograft model. Moreover, let-7c suppressed tumor growth and infiltration at the invasive front *in vivo*. Thus, these results indicated that let-7c plays a role in suppression of HNSCC development. miRNA-targeting therapies have been an area of intense interest to pharmaceutical companies, and let-7 is currently being developed as a potential miRNA replacement treatment for cancer. Although details of which cancer types are being investigated have not been disclosed, it will be interesting to see whether the delivery of miRNA mimetics can indeed have a therapeutic impact [35].

To clarify the mechanism of action of let-7c, we tried to elucidate the relation between let-7c and its targets: *IGF1R* and *HMGA2*. We demonstrated that the expression levels of *IGF1R* and *HMGA2* were significantly higher in HNSCC tumor tissues compared to non-tumor tissues, corresponding to downregulation of let-7c. Elevated expression of let-7c in HNSCC cells can downregulate *IGF1R* and *HMGA2* levels. Silencing of *IGF1R* and *HMGA2* inhibited cell growth, migration, and EMT, which indicates the oncogenic roles of these genes in HNSCC progression. The IGF signaling system has been implicated in the pathogenesis of various human cancers, including HNSCC [36]. The IGF1 receptor has emerged as a potential target molecule for cancer treatment [13, 37, 38]. Moreover, *IGF1R* has been reported to be essential for sustaining mesenchymal morphologies and plays an important role in regulating EMT in breast cancer [17]. *HMGA2* is abundant during embryogenesis and tumorigenesis, but is rarely present in normal adult tissues [39]. In addition, *HMGA2* expression can be detected in various cancers and is correlated with malignant degree and metastatic potential [16, 40, 41]. The oncogenic properties of *HMGA2* have been shown to influence a variety of biological processes, including tumor growth [42], DNA damage response [43], and EMT [44–46]. *HMGA2* has been associated with an aggressive cancer phenotype and induction of EMT by suppressing E-cadherin transcription in tongue cancer [18]. A recent report showed that cell proliferation was regulated by the *HMGA2-IGF2BP2* axis, which includes *IGF1R*, suggesting the presence of interactions between *HMGA2* and *IGF1R* [47]. Our findings and the literature demonstrate that let-7c-mediated regulation of *IGF1R* and *HMGA2* may affect the aggressiveness of HNSCC through EMT. Recent studies [48, 49] indicated the possibility that miRNA-based cancer therapy is to achieve specific, effective, and safe delivery of therapeutic miRNAs into cancer cells. Therefore, the results from this study offer a new therapeutic strategy for HNSCC treatment that warrants further investigation in future clinical studies. In conclusion, our study identifies let-7c as a novel tumor suppressor of HNSCC that inhibits cell proliferation, migration, and EMT through repression of *IGF1R* and *HMGA2* expression.

## MATERIALS AND METHODS

### Patient recruitment and sample collection

Paired tissue samples were collected from 15 patients with HNSCC who underwent surgery at the Department of Otorhinolaryngology-Head and Neck Surgery, Mie University Hospital, between 2012 and 2013. Clinical characteristics, including patient age, sex, TNM staging, and the status of HPV infection determined by p16 IHC staining are listed in Supplementary Table 1. Tissue specimens were immediately steeped in RNAlater (Ambion, Carlsbad, CA, USA) at 4°C for 24 h and subsequently stored at −80°C until analysis. This study was approved by the Mie University Graduate School of Medicine Ethical Committee (No. 2445). Written informed consent was obtained from each patient before the study.

### Cell culture

Non-malignant nasopharyngeal epithelial cell line NP69 was cultivated in keratinocyte serum-free medium (Gibco, Carlsbad, CA, USA). The HNSCC cell lines SAS, Ca9-22, and H0-1-u-1 (derived from tongue, gingiva, and mouth floor, respectively) were obtained from RIKEN BRC Cell Bank (Tsukuba, Japan). All these HNSCC cell lines were maintained in RPMI-1640 medium with 10% fetal bovine serum.

### miRNA plasmid transfection

The let-7c expression vector pCMV-MIR-let-7c and the pCMV-MIR vector were obtained from Origene (Rockville, MD, USA). Both vectors were monitored using green fluorescent protein. The let-7c expression vector and empty vector were transfected into SAS cells and Ca9-22 cells using FuGENE HD Transfection Reagent (Promega, Madison, WI, USA). Stable clones expressing let-7c (SAS-let-7c and Ca9-22-let-7c) or empty vector (SAS-empty and Ca9-22-empty) were obtained by G418 (Calbiochem, La Jolla, CA, USA; 600 μg/ml and 200 μg/ml, respectively) selection for 2 weeks. Three vectors (Origene), namely, pCMV6-control, pCMV6-IGF1R, and pCMV6-HMGA2, were used for the rescue study.

### Reverse transcription and RT-qPCR

Total RNA from cell lines was extracted using TRIzol reagent (Ambion). The integrity and quantity of total RNA were confirmed by gel electrophoresis and a NanoDrop 2000 spectrophotometer (NanoDrop, Wilmington, DE, USA). cDNA was synthesized from 500 ng RNA using a miScript Reverse Transcription Kit (Qiagen, Hilden, Germany). RT-qPCR was performed in duplicate using a miScript SYBR Green PCR kit (Qiagen) for samples with the miScript Universal Primer and the miRNA-specific forward primers. Expression levels of mRNA and miRNA were normalized by GAPDH and small nuclear RNA RNU6B, respectively. All amplifications were carried out in an ABI Step One Plus Real-time PCR System (Applied Biosystems, Singapore, Singapore).

### Western blotting

HNSCC tissues and cell lines were lysed for 10 min in ice-cold RIPA buffer (Cell Signaling Technology, Beverly, MA, USA) containing protease inhibitor cocktail tablets (Sigma-Aldrich, St. Louis, MO, USA) and then centrifuged at 14,000 g for 20 min at 4 °C. Supernatants were collected and stored at −80 °C. Protein concentrations were determined using a BCA (bicinchoninic acid) assay kit (Pierce, Tewksbury, MA, USA). SDS-treated proteins were separated on SuperSepAce 5–20% polyacrylamide gels (Wako Pure Chemical Industries, Osaka, Japan), and transferred to PVDF membranes. The PVDF membranes were then cut according to the molecular weight markers and membranes were stained using different antibodies. Primary antibodies against IGF1R-β (1:1000, #9750), HMGA2 (1:1000, #8179), E-cadherin (1:1000, #3195), and vimentin (1:1000, #5741) were obtained from Cell Signaling Technology. Immunoreactive bands were detected with a chemiluminescence reagent kit (ECL Prime; Amersham Bioscience, Arlington Heights, IL, USA) and quantified by densitometry with Image J software (NIH, Bethesda, MD, USA). GAPDH (1:2500; ab9485; Abcam, Cambridge, UK) was blotted on the same membrane as a loading control.

### Dual-luciferase reporter assay

A pmirGLO Dual-Luciferase miRNA Target Expression Vector was used for 3′-UTR luciferase assays (Promega). The putative target oncogenes of tumor suppressor let-7c were selected based on the TargetScan Human online database (Release 7.0: August 2015, http://www.targetscan.org/). There are three and seven predicted binding sites of let-7c in the 3′-UTRs of *IGF1R* and *HMGA2*, respectively. Predictions are ranked by their probability of conserved targeting (P_CT_) (Supplementary Table 2). To design the oligonucleotide sequences of *IGF1R* and *HMGA2*, we chose the binding sites of let-7c that achieved the highest P_CT_ scores. Oligonucleotide sequences of *IGF1R* and *HMGA2* containing the putative binding sites of let-7c were designed as shown in Supplementary Table 3. The double-stranded annealing products were inserted downstream of the firefly luciferase reporter in the pmirGLO dual-luciferase miRNA target expression vector. SAS cells were plated in 96-well plates for 24 h, then co-transfected with 50 ng of PCMV-MIR control vector or PCMV-MIR-let-7c vector and 100 ng dual-luciferase vector containing either wildtype or mutant 3′-UTR. At 24 h after transfection, the luciferase activity was measured with the Dual-luciferase Reporter Assay System (Promega).

### Colony formation and cell proliferation assay

Cell lines stably transfected with let-7c or empty vector (SAS-let-7c, Ca9-22-let-7c; SAS-empty, Ca9-22-empty) were seeded for colony formation in 6-well plates at 500 cells per well. After 9 days, colonies were scored using a microscope by adding Giemsa (Merck, Darmstadt, Germany). Colonies were counted only if the diameter of a single colony was larger than 75 μm in diameter. Each assay was performed in triplicate on two independent occasions. For cell proliferation assays, growth curves of cell lines stably transfected with let-7c or empty vector (SAS-let-7c, Ca9-22-let-7c; SAS-empty, Ca9-22-empty) were monitored by cell counting. Transfected cells were seeded at a density of 5 × 10^4^ cells/mL per well in 6-well plates. The cells were counted using the TC 20 Automated Cell Counter (Bio-Rad, Hercules, CA, USA).

### Migration assay

Cell migration assays were performed using the CytoSelect Cell Migration Assay Kit (8-μm pore membrane filter; Colorimetric Format; Cell BioLabs, San Diego, CA, USA). To study the role of let-7c in tumor biology, cells were suspended in serum-free medium (0.15 × 10^6^ transfected cells/well) and placed in the upper chamber of the migration plate, and medium containing 10% FBS was placed in the lower chamber. After incubation for 24 h and removal of non-migratory cells, cells that migrated through the filter were stained. Each filter with migratory cells was extracted, and relative migration level was quantified by measuring absorption at 595 nm using a microplate reader (Model 680, Bio-Rad).

### IHC and ICC study

For IHC analysis, standard immunoperoxidase methods were used to examine the distribution of IGF1R and HMGA2 in HNSCC tissues and adjacent non-tumor tissues. After deparaffinization and rehydration, antigen was retrieved in 5% urea buffer by microwave heating for 5 min, and then inctubated in 1% H_2_O_2_ for 30 min to block endogenous peroxidase activity. Sections of 6-μm thickness were incubated overnight at room temperature with the following antibodies: rabbit monoclonal IGF1R-β (1:200) and rabbit monoclonal anti-HMGA2 (1:100). Nuclei were counterstained with hematoxylin, with the exception of HMGA2 since its immunostaining is located in nuclei. For ICC analysis, cells stably transfected with let-7c were fixed with 4% (v/v) formaldehyde in phosphate buffered saline (PBS) for 10 min at room temperature and washed three times with PBS. The cells were treated with 1% (v/v) Triton X-100 for 20 min, then incubated with 5% (w/v) skim milk for 60 min at room temperature. Immunofluorescence was performed by overnight incubation at room temperature with rabbit monoclonal IGF1R-β (1:1600), rabbit monoclonal anti-HMGA2 (1:400), rabbit monoclonal E-cadherin (1:200), and vimentin (1:100), all obtained from Cell Signaling Technology. The cells were then incubated with fluorescent secondary antibodies (Alexa Fluor 594–labeled goat anti–rabbit IgG, 1:400 each, Molecular Probes) for 2 h. Nuclei were stained with DAPI (SouthernBiotech, Birmingham, AL, USA) and the stained cells were examined under a fluorescence microscope (BX53; Olympus, Tokyo, Japan). The number of positively staining cells was analyzed using ImageJ software (ver. 1.48).

### Nude mice xenograft model and hematoxylin & eosin staining

Four-week-old male BALB/c athymic nu/nu mice (Japan SLC, Hamamatsu, Japan, weight range 16–19 g) were used in these experiments and maintained at the Institute of Laboratory Animals at Mie University. All animal experiments were performed according to the Mie University guidelines for laboratory animals (approval No. 26-19). A total of 8 animals were housed in ventilated cages under a 12 h dark/light cycle at constant humidity and temperature. Animals were permitted free access to sterile water and standard laboratory chow. Subcutaneous xenografts were established by inoculating 2 × 10^6^ let-7c stably transfected SAS cells into the left flank, or an equal number of empty vector–transfected cells into the right flank. The tumor volume was measured with a caliper (model 530-312; range 0–150 mm; Mitutoyo, Kawasaki, Japan) and calculated using the following formula: tumor volume (mm^3^) = length × width^2^/2, where length and width were the longer and shorter dimensions of the tumor, respectively. Seventeen days after the implantation, the mice were sacrificed and the tumors were removed and weighed. Tumors were fixed in 4% formaldehyde. Primary antibodies against IGF1R-β (1:200), HMGA2 (1:100), E-cadherin (1:100), and vimentin (1:100) were used for IHC analysis.

## SUPPLEMENTARY MATERIALS FIGURES AND TABLES



## Abbrevations

HNSCC: Head and neck squamous cell carcinoma
*IGF1R*: insulin-like growth factor 1 receptor
*HMGA2*: high mobility group AT-hook 2
EMT: epithelial–mesenchymal transition
3′-UTR: 3′-untranslated region; HPV: human papilloma virus
