# Supplementary material for: Let-7c inhibits migration and epithelial–mesenchymal transition in head and neck squamous cell carcinoma by targeting IGF1R and HMGA2
Source: Oncotarget. 2018 Jan 2;9(10):8927–40. doi: 10.18632/oncotarget.23826 (PMC5823619; doi:10.18632/oncotarget.23826)
Supplement: Supplementary file 1 [file oncotarget-09-8927-s001.pdf]

# Let-7c inhibits migration and epithelial–mesenchymal transition in head and neck squamous cell carcinoma by targeting *IGF1R* and *HMGA2*

## SUPPLEMENTARY MATERIALS

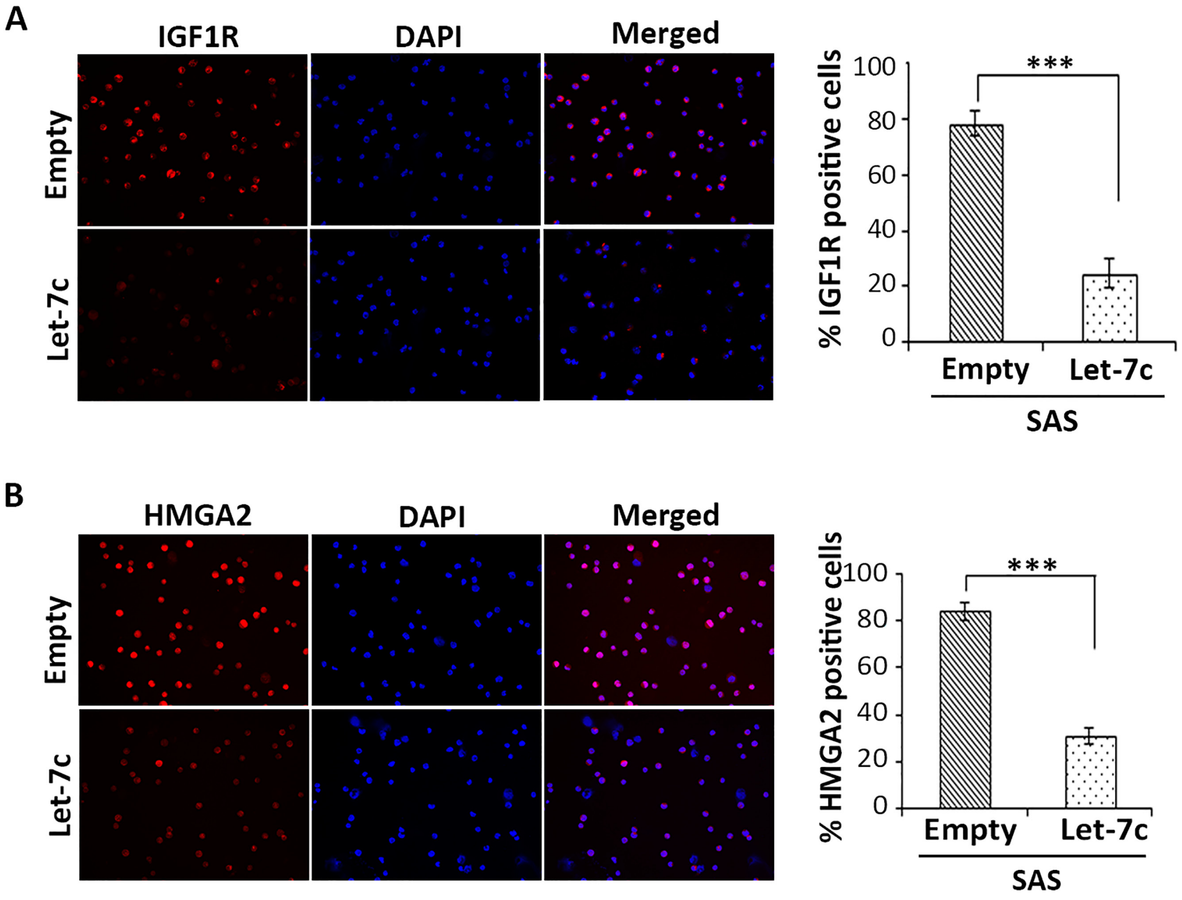


**Supplementary Figure 1: Protein levels of IGF1R and HMGA2 in stably transfected HNSCC cells.** The levels of (**A**) IGF1R and (**B**) HMGA2 in let-7c–expressing SAS cells were determined using ICC staining. Numbers of positively staining cells and total cell number (determined by DAPI staining of nuclei) were analyzed using ImageJ software, and the percentage of positively stained cells was calculated for each sample ($n = 3$) in three areas. *P*-values were calculated using the Student's *t*-test. $^{***}P < 0.001$.

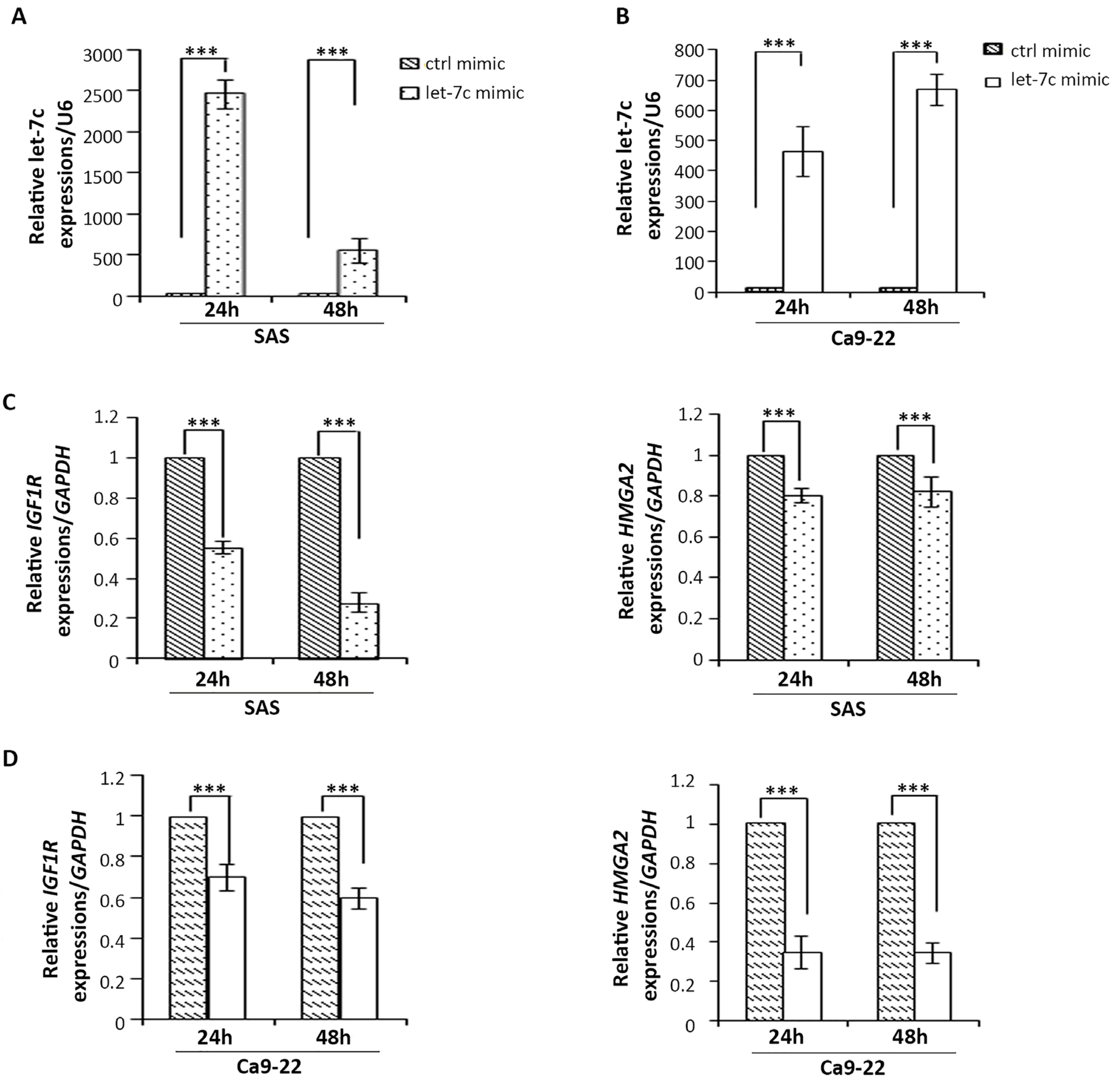


**Supplementary Figure 2: Transient transfection of let-7c mimic in HNSCC cell lines inhibits expression of both *IGF1R* and *HMGA2*.** (**A, B**) Expression levels of let-7c and (**C, D**) *IGF1R* and *HMGA2* mRNAs were examined in SAS and Ca9-22 cell lines, respectively, which were transiently transfected with either control miRNA or let-7c mimic. *P*-values were calculated using the Student's *t*-test. $^{***}P < 0.001$.

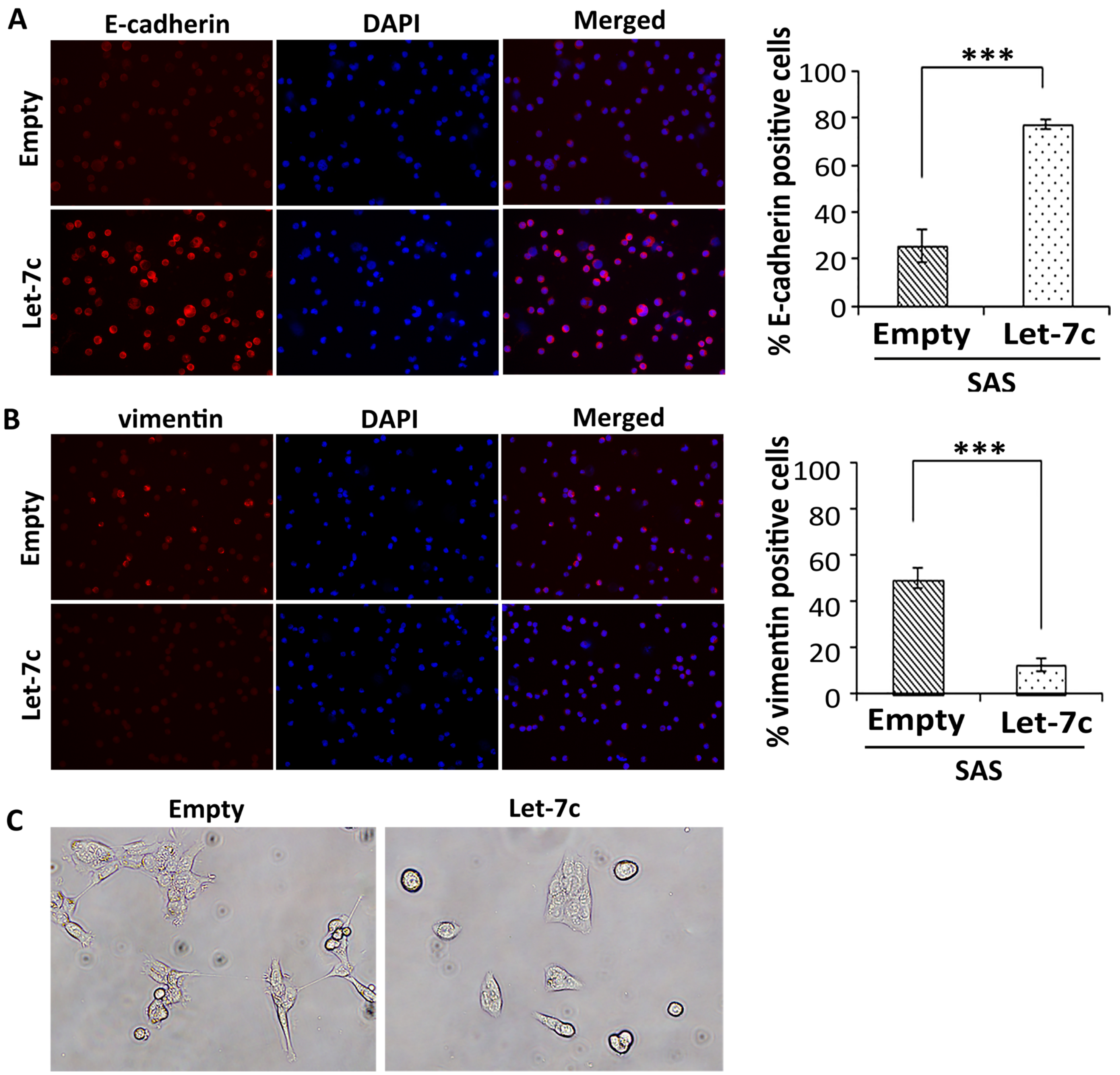


**Supplementary Figure 3: Protein levels of E-cadherin and vimentin in stably transfected HNSCC cells.** (**A**) E-cadherin and (**B**) vimentin levels in let-7c–expressing SAS cells were determined using ICC. Numbers of positively staining cells and total cell number (determined by DAPI staining of nuclei) were analyzed using ImageJ software, and the percentage of positively stained cells was calculated for each sample ($n$ = 3) in three areas. $P$-values were calculated using the Student's $t$-test. $^{***}P < 0.001$. (**C**) Morphological changes in let-7c–expressing SAS cells compared to empty control cells.

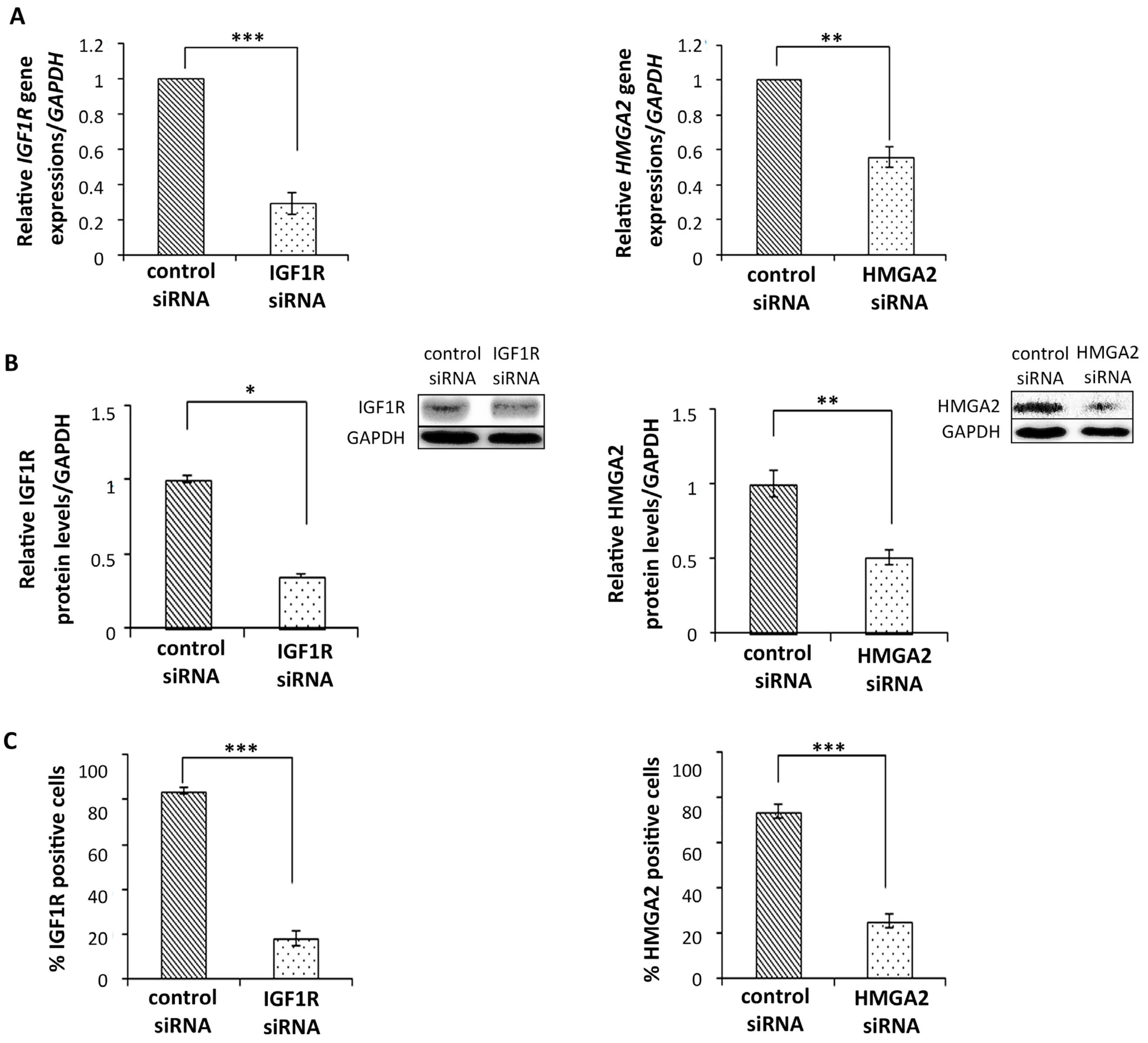


**Supplementary Figure 4: Evaluation of *IGF1R* and *HMGA2* mRNA expression using corresponding siRNAs.** (**A**) *IGF1R* and *HMGA2* mRNA levels were examined by qRT-PCR, and IGF1R and HMGA2 protein levels were determined by (**B**) western blot and (**C**) ICC in HNSCC cell line SAS. *P*-values were calculated using the Student's *t*-test. $^{**}P < 0.01$; $^{***}P < 0.001$.

**Supplementary Table 1: Clinicopathologic characteristics of HNSCC patients**

| Group | No. of cases (%) | HPV status p16-positive (%) |
|---|---|---|
| Sex | | |
| Male | 14 (93.3%) | 8 (66.7%)** |
| Female | 1 (6.7%) | 0 (0 %) |
| Age | | |
| Range | 53–80 | |
| Mean ± SD | 70.7 ± 6.8 | |
| Sites | | |
| Maxillary sinus | 1 (6.7%) | 1 (100 %) |
| Oral cavity | 3 (20.0%) | 1 (50.0%)* |
| Oropharynx | 4 (26.7%) | 3 (75.0%) |
| Hypopharynx | 5 (33.3%) | 3 (60.0%) |
| Larynx | 2 (13.3%) | 0 (0 %)* |
| TNM stage | | |
| I | 0 (0.0%) | – |
| II | 3 (20.0%) | 2 (100%)* |
| III | 3 (20.0%) | 2 (66.7%) |
| IV | 9 (60.0%) | 4 (50.0%)* |

*One case had no information of HPV status.
**Two cases had no information of HPV status.
Mouse monoclonal anti-p16 antibody (Santa Cruz Biotechnology, Santa Cruz, CA, 1:200), the secondary antibody conjugated with HRP, and DAB substrate for peroxidase (Vector Laboratories) were used for IHC.

**Supplementary Table 2: Let-7c binding sites of each target genes (IGF1R and HMGA2)**

| **Human IGF1R 3′-UTR (let-7c binding sites in bold)** | | | |
|---|---|---|---|
| Position | Predited consequential paring of target region | Site type | $P_{CT}$ |
| 2619-2626nt | CCCCCAAACAUUUAU**CUACCUCA** | 8mer | 0.98 |
| 6661-6667nt | UUUGCCAGAGUUUGU**CUACCUC**U | 7mer-m8 | 0.94 |
| 99-105nt | UUCACAAGCCUCCUG**UACCUCA**G | 7mer-A1 | 0.9 |
| **Human HMGA2 3′-UTR (let-7c binding sites in bold)** | | | |
| Position | Predited consequential paring of target region | Site type | $P_{CT}$ |
| 21-28nt | GCCAACGUUCGAUUU**CUACCUCA** | 8mer | 0.94 |
| 2521-2527nt | AAUCAAAACACACUA**CUACCUC**U | 7mer-m8 | 0.91 |
| 2541-2547nt | UCUUAAGUCCCAGUA**UACCUCA**U | 7mer-A1 | 0.89 |
| 1668-1674 | UUGACUUGCAAAGAC**CUACCUC**C | 7mer-m8 | 0.86 |
| 1256-1262nt | CCCCACUACUCAAUA**CUACCUC**U | 7mer-m8 | 0.86 |
| 1619-1625nt | UACCCUCCAAGUCUG**UACCUCA**A | 7mer-A1 | 0.83 |
| 1107-1113nt | GACCUGAAUACCACU**UACCUCA**A | 7mer-A1 | 0.81 |

TargetScan Human online database (Release 7.0: August 2015, http://www.targetscan.org/)

**Supplementary Table 3: Oligonucleotide sequences of *IGF1R* and *HMGA2***

| Oligonucleotides | Sequence |
|---|---|
| IGF1R WT sense | 5′-AAACTAGCGGCCGCTAGTCCCCCAAACATTTATCTACCTCAT-3′ |
| IGF1R WT antisense | 5′-CTAGATGAGGTAGATAAATGTTTGGGGGACTAGCGGCCGCTAGTTT-3′ |
| IGF1R MT sense | 5′-AAACTAGCGGCCGCTAGTCCCCCAAACATTTATGATGGAGTT-3′ |
| IGF1R MT antisense | 5′-CTAGAACTCCATCATAAATGTTTGGGGGACTAGCGGCCGCTAGTTT-3′ |
| HMGA2 WT sense | 5′-AAACTAGCGGCCGCTAGTGCCAACGTTCGATTTCTACCTCAT-3′ |
| HMGA2 WT antisense | 5′-CTAGATGAGGTAGAAATCGAACGTTGGCACTAGCGGCCGCTAGTTT-3′ |
| HMGA2 MT sense | 5′-AAACTAGCGGCCGCTAGTGCCAACGTTCGATTTGATGGAGTT-3′ |
| HMGA2 MT antisense | 5′-CTAGAACTCCATCAAATCGAACGTTGGCACTAGCGGCCGCTAGTTT-3′ |
